# A novel *PTCH1* mutation in basal cell nevus syndrome with rare craniofacial features

**DOI:** 10.1038/s41439-019-0047-9

**Published:** 2019-04-02

**Authors:** Yuka Murata, Hiroshi Kurosaka, Yasuhisa Ohata, Tomonao Aikawa, Sosuke Takahata, Katsunori Fujii, Toshiyuki Miyashita, Chisato Morita, Toshihiro Inubushi, Takuo Kubota, Norio Sakai, Keiichi Ozono, Mikihiko Kogo, Takashi Yamashiro

**Affiliations:** 1 Department of Orthodontics and Dentofacial Orthopedics, Osaka University Graduate School of Dentistry, Suita, Japan; 2 The First Department of Oral and Maxillofacial Surgery, Osaka University Graduate School of Dentistry, Suita, Japan; 3 Department of Pediatrics, Osaka University Graduate School of Medicine, Suita, Japan; 4 Department of Pediatrics, Graduate School of Medicine, Chiba University, Chiba, Japan; 5 Department of Molecular Genetics, Kitasato University Graduate School of Medical Sciences, Sagamihara, Japan; 6 Child Healthcare and Genetic Science Laboratory, Division of Health Sciences, Osaka University Graduate School of Medicine, Suita, Japan

**Keywords:** Disease genetics, Development

## Abstract

Basal cell nevus syndrome (BCNS) is a rare, multisystem, autosomal dominant disorder that is characterized by various phenotypes, including multiple basal cell carcinomas of the skin, odontogenic keratocysts of the jaws, and occasionally cleft lip and/or palate. In this report, we describe a 6-year-old Japanese girl with a novel heterozygous nonsense mutation in *PTCH1* who exhibited rare craniofacial phenotypes, such as oligodontia and a short-tooth root.

Basal cell nevus syndrome (BCNS) (OMIM #109400), also known as Gorlin syndrome, is a rare, multisystem, autosomal dominant disorder with various phenotypes, including multiple basal cell carcinomas of the skin, palmar, and/or plantar pits and bifid ribs^1^. Typical craniofacial phenotypes are also associated with BCNS, such as keratocystic odontogenic tumor (KCOT), Falx calcification, macrocephaly, hypertelorism, hydrocephalus, and cleft lip/palate, which occur at different frequencies^1^. Based on these characteristic symptoms, BCNS can be discovered during orofacial examination. The prevalence of BCNS has been reported to be approximately 1 per 235,800 in the Japanese population, with an equal frequency in males and females^1^, though the reported frequency varies among studies^2^. Although most cases of BCNS are identified in patients aged between 17 and 35 years, it is sometimes diagnosed in very young children^3^. Genetic testing can be applied to distinguish BCNS from other syndromes and to obtain a clear prognosis.

Three different genes, *PTCH1* (OMIM *601309)^4^, *PTCH2* (OMIM *603673)^5^, and *SUFU* (*607035)^6^, have been identified as being responsible for BCNS, with most mutations occurring in the *PTCH1* gene. Despite no genotype–phenotype correlation among the various mutations in *PTCH1*^7^, patients with *SUFU* mutations exhibit a high frequency of medulloblastoma but do not develop KCOT^8^. Treatment of BCNS includes an individualized multidisciplinary therapeutic approach. Keratocysts usually require surgical excision, and aggressive basal cell carcinomas require complete eradication. Surgical excision is supplemented by a number of other possible treatments, including cryotherapy, laser treatment, and photodynamic therapy^9^. With regard to craniofacial defects such as cleft lip/palate, which occur in 2–9.1% of cases, chelioplasty, palatoplasty, and subsequent orthodontic treatment are generally required. Approximately, 90% of patients develop KCOT at various ages, typically requiring enucleation to prevent severe tooth disruption and jaw fracture^1,10^. Because of the widely divergent expressivity of the BCNS phenotype, continuous investigations and reports of individual cases with physical and genetic information are crucial for expanding our knowledge.

The present patient was a 6-year-old Japanese girl referred to the Osaka University Dental Hospital Department of Orthodontics for the management of malocclusion. She exhibited several symptoms, including ocular hypertelorism (Fig. 1a), bilateral cleft lip and palate (Fig. 1b), and palmar pits (Fig. 1c), with a history of cheiloplasty, palatoplasty, and surgical excision of cardiac fibroma. Craniofacial examination revealed a typical facial profile for cleft lip and palate, with midfacial deficiency and upper lip retrusion. Ensuing cephalometric analysis showed a long-cranial base and a normal jaw-base relationship (Fig. 1d, e). Intraoral examination showed an anterior open bite with bilateral cleft lip and palate, a narrow upper intercanine width and palatally inclined upper incisors. Multiple KCOTs on both sides of the mandible, one around the lower left canine, and another around the lower right second molar were observed by panoramic radiography (Fig. 1f). Interestingly, additional dental anomalies were found, such as oligodontia (the upper lateral incisors, lower left first premolar, and upper and lower second premolars) and a truncated root of the lower first molars. None of her relatives exhibited any of these symptoms. Based on these clinical findings, we suspected that this patient had BCNS, and we suggested that the patient’s family undergo genetic counseling. After explaining all the risks and benefits of genetic testing during counseling, the parents of the patient decided to receive genetic testing for BCNS. Genetic analysis approved by the Institutional Review Board of Chiba University and Kitasato University was planned to determine the gene mutation in this patient. After written informed consent had been obtained, genomic DNA was extracted from peripheral blood, and the genomic DNA in the samples was amplified with primers targeting all exons of the *PTCH1* gene, as described previously^11^. The amplified products were gel-purified using a QIAex II Gel Extraction Kit (Qiagen, Hilden, Germany) and cycle sequenced in both directions using a BigDye Terminator v3.1 Cycle Sequencing Kit (Applied Biosystems, Foster City, CA, USA), and the sequences were analyzed using a 3130 Genetic Analyzer (Applied Biosystems, Foster City, CA, USA). This analysis revealed a heterozygous nonsense mutation in exon 8 of *PTCH1* (NM_000264.4:c.1111C > T, p. Q371*) (Fig. 2a, b). Considering the almost complete penetrance of this disorder and the fact that neither parent presented the BCNS phenotype, the patient’s mutation is suggested to be de novo. The mutation detected in this patient was not found in the 1000 Genomes or Exome Aggregation Consortium database.

**Fig. 1:**
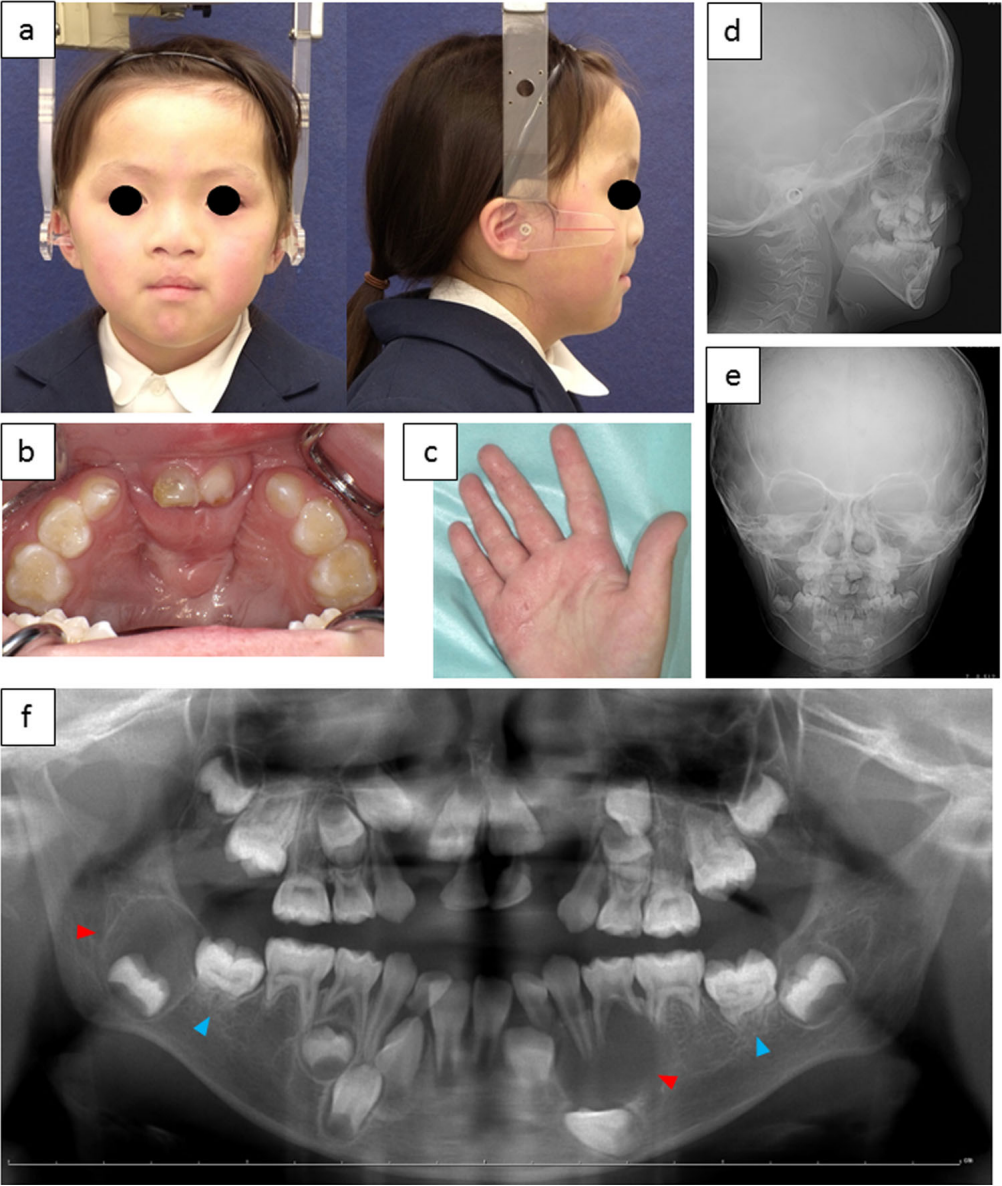
Clinical features of the present BCNS patient. **a** Facial photographs. **b** Cleft lip and palate. **c** Palmar pits. **d**, **e** Cephalometric radiograph. **f** Panoramic radiograph. Red arrows show multiple cystic radiolucent areas, and blue arrows show root hypoplasia

**Fig. 2:**
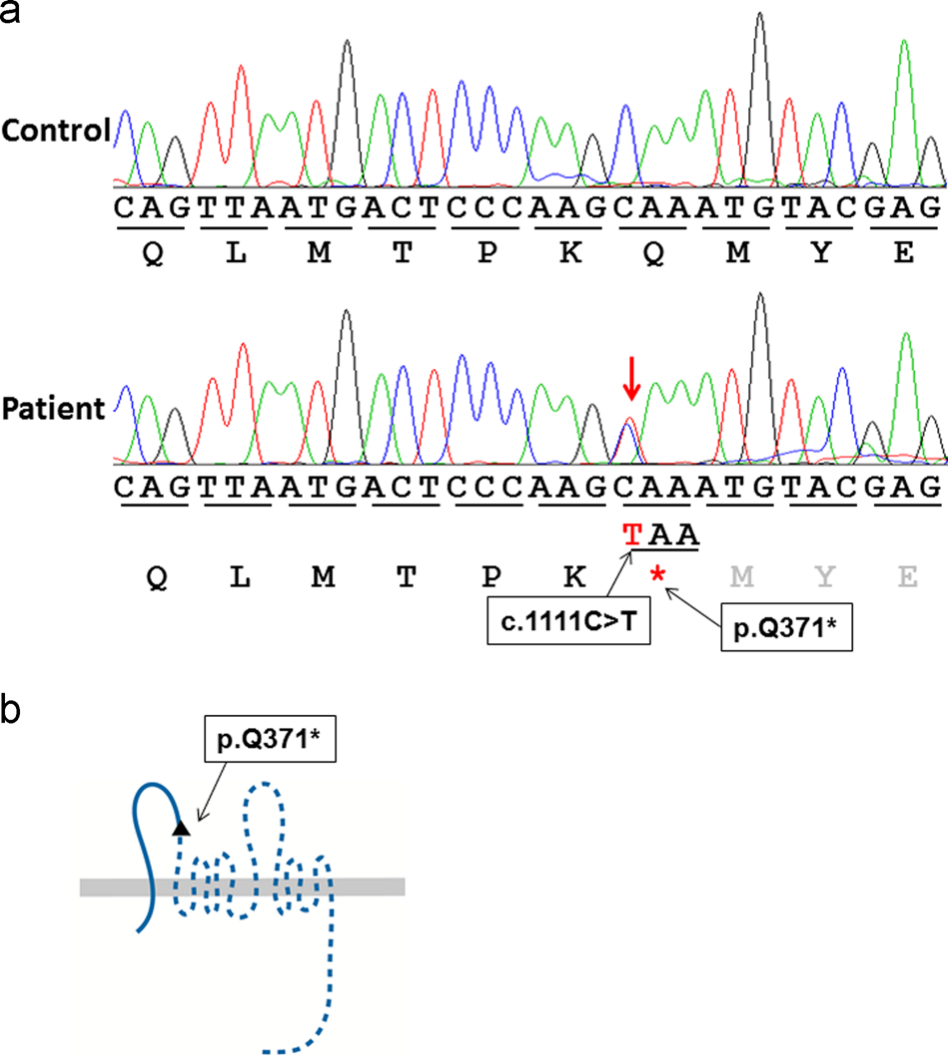
Mutation findings. **a** Sequence analysis. DNA sequence chromatograms showing the heterozygous nonsense mutation (red arrowhead) in exon 8 of *PTCH1* (LRG_515t1:c.1111C > T). **b** Localization of the *PTCH1* mutation on a schematic image of protein patched homolog 1. The black arrowhead indicates the region of the nonsense mutation in the patched protein

The patient underwent enucleation of KCOTs at the age of 6, and histopathological examination also confirmed the diagnosis. She is now awaiting tooth eruption and subsequent correction of the malocclusion by orthodontic treatment. The present case is one example of BCNS diagnosed at a relatively young age by orofacial examination and demonstration of *PTCH1* mutation.

PTCH1 is one of the best studied receptors of the Sonic Hedgehog (SHH) ligand. In the absence of ligand, PTCH1 functions as a repressor of SHH signaling^4^. During embryogenesis, SHH signaling plays critical roles in various tissues and organs, and thus, disturbance of this signaling pathway results in a variety of defects^12^. Because PTCH1 plays a repressive role in SHH signaling, loss of *PTCH1* function usually results in elevated activity of the pathway. In some animal experiments, craniofacial defects, including congenital cranial dysinnervation disorders, hypertelorism, cleft lip, and palate due to *PTCH1* loss of function mutations have been reported, in accord with the phenotype of the present patient^13–15^. Cleft lip and palate is known to be a multifactorial condition, and from the high incidence in BCNS, *PTCH1* mutation is suggested to be one of the contributing genetic causes in these patients. Indeed, some GWAS studies report that the incidence of *PTCH1* mutation is high in the facial cleft population^16^. Interestingly, some tooth phenotypes that have not been previously reported in BCNS, such as oligodontia and short root, were observed in the present case. Shh signaling play critical roles during tooth development^17^, and previous studies have shown that disturbing Shh signaling results in tooth development arrest and mispatterned dentition^18^. Additionally, by affecting cell proliferation in the tissue called the Hertwig epithelial root sheath, which is critical for root elongation, time- and tissue-specific disruption of Shh signaling results in short-tooth roots^19,20^. However, the mechanism of tooth root development has not been fully elucidated, and further research will be required to explain the possible mechanism of the truncated root in BCNS. Nevertheless, considering the requirement for orthodontic treatment, tooth anomalies should be taken into account if they exist in BCNS patients. The literature suggests variations in the phenotype of BCNS patients and that *SUFU* mutation does not result in KCOT^8^. This might indicate a tissue-specific requirement for different genes, even though both *PTCH1* and *SUFU* regulate the same signaling pathway. In this study, we report one case of BCNS with a novel mutation in *PTCH1* that resulted in a rare craniofacial phenotype combination. Additional reports of cases such as this will be necessary to increase our knowledge and investigate possible genotype–phenotype correlations in BCNS.

## Data Availability

The relevant data from this Data Report are hosted at the Human Genome Variation Database at 10.6084/m9.figshare.hgv.2546.
